# Severe episodic memory impairment after strategic infarct: A case report

**DOI:** 10.1590/1980-57642016dn11-040017

**Published:** 2017

**Authors:** Francisco Wilson Nogueira Holanda, Katie Moraes de Almondes, Rodrigo Alencar e Silva

**Affiliations:** 1 Master's student on the Postgraduate Program in Psychology, Federal University of Rio Grande do Norte, RN, Brazil.; 2 Associate Professor at the Department of Psychology and on the Postgraduate Program in Psychobiology, Federal University of Rio Grande do Norte, RN, Brazil.; 3 Neurologist at the University Hospital Onofre Lopes, Federal University of Rio Grande do Norte, RN, Brazil.

**Keywords:** memory, strategic infarct, neuropsychological assessment, stroke, memória, infarto estratégico, avaliação neuropsicológica, acidente vascular encefálico

## Abstract

Brain infarcts located in strategic regions often result in cognitive impairment. Based on a case study, this paper describes unusual and specific clinical and neuropsychological features of a strategic ischemic lesion in the left medial temporal lobe (MTL) structures. Taken together with the literature data, the case illustrates that a unilateral strategic infarct in MTL structures may result in severe impairment of episodic memory (EM), which refers to the ability to encode and retrieve personal experiences, including information about the time and place of an event and detailed description of the event itself. The preservation of other cognitive functions, the severe functional impairment, and the type of visual-verbal deficit in a left-sided lesion were identified as singular features of the case. The current case supports the critical role of the MTL structures in EM formation.

## RESUMO.

Infartos cerebrais localizados em regiões estratégicas comumente resultam em comprometimento cognitivo. Baseado em um estudo de caso, este artigo descreve características clínicas e neuropsicológicas incomuns e específicas de uma lesão isquêmica estratégica nas estruturas mediais do lobo temporal esquerdo. Tomado em conjunto com os dados da literatura, este caso ilustra que uma lesão isquêmica estratégica unilateral em estruturas do lobo temporal medial resulta no comprometimento da memória episódica, a qual se refere à habilidade de armazenar e recuperar experiências pessoais, o que inclui informações sobre o tempo e o local do evento e a descrição detalhada do próprio evento. A preservação de outras funções cognitivas, o grave comprometimento funcional e a modalidade de déficit visual-verbal em uma lesão à esquerda foram apontados como características singulares do caso. Este estudo de caso corrobora o papel crítico das estruturas do lobo temporal medial na formação da memória episódica.

## INTRODUCTION

The occurrence of strategically located ischemic vascular lesions in brain areas critical for cognition and behavior (e.g., associative, limbic, paralimbic circuitry) is referred to as strategic infarcts (SI).^1^ Regions such as the angular gyrus, the medial part of the frontal lobes, the medial part of the temporal lobe, the thalamus, and the caudate nucleus are the most common sites of strategic infarcts.^2^^,^ 3 SI are of great interest to cognitive neurology and clinical neuropsychology due to the possibility of studying the relationship between cognitive impairments and underlying brain damage (i.e., brain structure-function correlation).

SI are often associated with the gradual or abrupt development of mild vascular cognitive impairment and even dementia.^4^^,^ 5 Depending on the location, extent and severity of the lesions, patients may present varying degrees and types of cognitive impairment, such as memory deficits.^6^ Episodic memory (EM), which refers to the ability to encode and retrieve daily personal experiences,^7^ is one of the most discussed memory systems. Clinical features of impairment in EM may be observed in lesions that are more limited to the medial temporal lobe (MTL) region.^8^ However, data concerning alterations in EM associated with SI in the MTL structures are lacking.

This paper reports a case of severe episodic memory impairment as a result of focal ischemic lesion in the left MTL structures, contrasting and discussing unusual and specific clinical and neuropsychological features: (1) impairment of verbal and visual mnemonic modalities in a left-sided lesion; (2) preservation of other cognitive functions; and (3) severe functional impairment. Case reports with these characteristics are uncommon in Brazilian scientific literature, and highlights the importance of the brain structure-function correlation, a fundamental cornerstone of cognitive neurology and neuropsychology.

## CASE REPORT

JE, a 68-year-old right-handed male with 10^th^-grade education was referred by a neurologist for neuropsychological evaluation in December 2016 due to forgetfulness which emerged after January of the same year. In January 2016, he had suffered an ischemic stroke of cardioembolic etiology. JE had a medical history of hypertension and chronic atrial fibrillation. According to JE and his wife, before the stroke he was normally able to manage his home activities and job tasks as a machine mechanic. However, after the stroke, the patient had severe difficulty organizing his finances and could no longer work or drive. His family perceived memory deficits such as forgetting new events, commitments, asking about people who died recently, and repeatedly asking the same question. JE also presented disorientation in time and new places.

**Figure 1:**
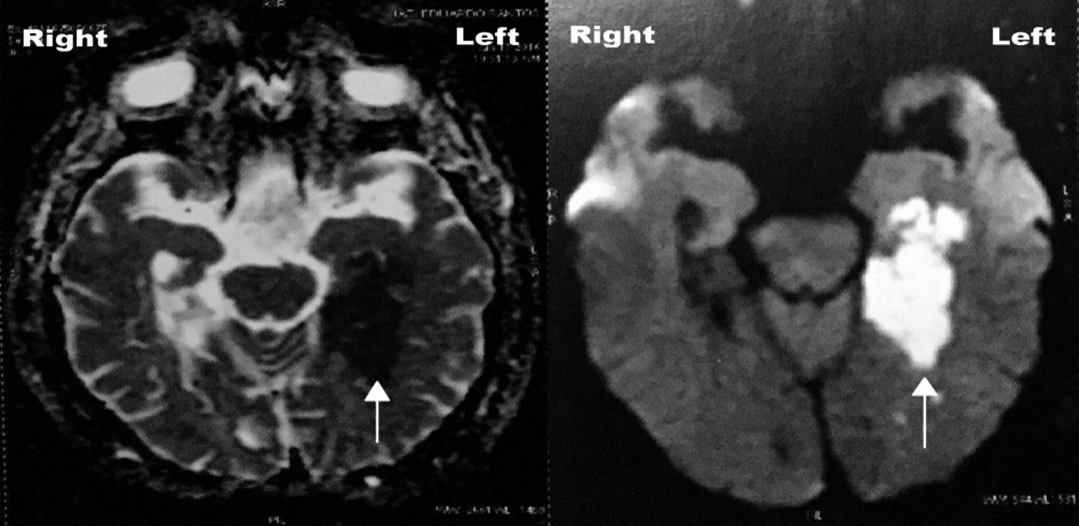
Axial images show (indicated by arrows) restricted diffusion in left parahippocampal and hippocampal regions.

On medical evaluation, laboratory test results were within normal ranges (including blood count, comprehensive metabolic panel, thyroid-stimulating hormone, vitamin B12 and venereal disease research laboratory). A magnetic resonance imaging scan of the brain performed in January 2016 indicated an ischemic lesion affecting the left parahippocampal gyrus, hippocampus, and anterior portion of the middle and inferior temporal gyrus. There were some additional tiny lesions of the white matter not exceeding possible typical age-related changes (Figure 1). At the time of the neurologist's observation, JE scored 23/30 on the Mini-Mental State Examination (MMSE), with deficits mainly on the subitems of time, place, and delayed recall. This demonstrated a marked decline of orientation and EM. On the Montreal Cognitive Assessment (MoCA), JE's score (18/30) was mainly attributed to errors in memory and orientation subtests. A diagnostic hypothesis of neurodegenerative dementia (e.g., Alzheimer's disease dementia) was excluded. There was evidence of a stroke temporally related to the onset of memory impairment, as well as lack of insidious onset of the decline. Therefore, the diagnosis of probable Alzheimer's disease dementia and other neurodegenerative dementias was not applicable.^9^

The patient underwent a comprehensive neuropsychological assessment to evaluate memory impairment and other functions (Table 1). Overall, JE's performance showed severe EM impairment in verbal and visual modalities indicated by delayed recall measures of the Rey Auditory Verbal Learning Test (RAVLT) (0/15) and the Rey-Osterrieth Complex Figure Test (RCFT) (3/36) with scores below the 5^th^ percentile. The Memory Subscale from the Dementia Rating Scale (DRS – 2) also indicated impaired performance (10/25, < 5^th^ percentile). He underperformed on the RAVLT Word Recognition list, which may indicate an inability to encode and consolidate new episodic contents. Category fluency (animals form) and Vocabulary (WAIS III) tests indicated preserved semantic memory (50^th^ percentile). JE had an appropriate strategy for visuospatial construction, making few mistakes on the ROCF Copy test. Regarding language processes such as naming and comprehension, there were no indications of impairment according to results on the Boston Naming Test (short version) and Token Test, respectively.

**Table 1:** Patient's neuropsychological background.

Neuropsychological background			Score
**Global cognitive status**	Montreal Cognitive Assessment		18/30*
	Clock Drawing Test		4/5
	Dementia Rating Scale (DRS – 2)	• Total	120/144*
		• Attention	36/37
		• Initiation/Perseveration	33/37
		• Construction	6/6
		• Conceptualization	35/39
		• Memory	10/25*
**Attention and executive functions**	Frontal Assessment Battery		14/18
	Digit Span Test	• Span (forward)	5/9
		• Total score (forward)	40/144
		• Span (backward)	5/8
		• Total score (backward)	30/112
	Corsi Block-tapping Test	• Span (forward)	6/9
		• Total score (forward)	48/144
		• Span (backward)	5/8
		• Total score (backward)	30/112
	Five Digits Test	• Reading (time)	60*
		• Counting (time)	51*
		• Choosing (time)	78*
		• Shifting (time)	96*
		• Inhibition Index	18
		• Flexibility Index	36
	Verbal fluency Test (FAS form)		23
**Memory (episodic and semantic)**	Rey Auditory Verbal Learning Test	• ΣA1A5	36/75
		• A7	0/15*
		• Recognition	–13*
	Rey Complex Figure Test (30 min. recall)		3/36*
	Verbal fluency Test (animals form)		14
	Vocabulary (WAIS III)		49/64
**Language (naming and comprehension)**	Boston Naming Test (short version)		13/15
	Token Test		31/36
**Visuo-construction**	Rey Complex Figure Test (copy)		33/36
**Functionality and behaviorial changes**	Functional Activities Questionaire		11/30
	Geriatric Depression Scale (GDS – 15)		6/15
	Neuropsychiatric Inventory Questionnaire	• Depression/dysthimia	4/12
		• Irritability	2/12

* Punctuations of cognitive tests indicated impaired performance according to published normative data for standardized tests.

There was no evidence of executive impairment in general. On the Five Digit Test, which is a numeric-Stroop paradigm applying four steps (Reading, Counting, Choosing and Shifting) that evaluates processes such as processing speed, selective attention, inhibitory control and cognitive flexibility, JE had scores indicative of clinical impairment (< 5^th^ percentile). However, considering the indices of Inhibition and Flexibility (interference scores that subtract Reading time from Choosing and Shifting time, which minimize the influence of processing speed on executive performance), there was no indication of impairment in these measures (50^th^ percentile). This discrepancy can be explained by the decreased processing speed observed in the patient, and not by a deficit in superior/executive functioning. This is corroborated by the fact that the patient was slow to perform the tests, but did so without errors, and due to the fact that he had adequate performance in all other tests of executive functioning.

## DISCUSSION

The main finding of this report was that an isolated left unilateral first-ever ischemic stroke confined to the MTL structures manifesting as an anterograde EM impairment. JE was unable to consolidate new episodic verbal and visual content, events and information, while other cognitive functions were found to be intact (except for slow processing speed). The capacity to consciously recollect information acquired at a certain time and place is referred to as EM.^10^ Tulving^11^ described EM as memory of personally experienced events or remembering what happened, where and when. Normal EM functioning involves a set of interconnected brain circuits, especially the MTL region. MTL represents a system of highly related structures, including the hippocampal formation (the dentate gyrus, CA1, CA2, CA3 subfields, and the subiculum) and the collectively denominated parahippocampal region (adjacent cortical areas, namely, entorhinal, perirhinal, and parahippocampal cortices).^7^

In the MTL, information originating from neocortical areas is processed through a hierarchical network represented by: (1) the perirhinal and parahippocampal cortices; (2) the entorhinal cortex; and (3) the hippocampal formation itself. The outputs of hippocampal processing encompass feedback connections successively back (i.e., towards the entorhinal cortex, then perirhinal and parahippocampal cortices) to neocortical areas from which the inputs to the MTL were generated.^12^ EM is mainly contingent on hippocampal and parahippocampal processing that facilitates consolidation of episodic information (e.g., new verbal and visual contents, spatial context, object recognition) from short-term to long-term memory.^13^ In this mediation, the hippocampal system and related structures then slowly transfer information into the neocortical storage system. Notably, other areas beyond the MTL, such as the dorsomedial nucleus of the thalamus, mammillary bodies, amygdala, and the basal forebrain also play a role in EM functioning.^14^ Due to the complex functioning of the MTL circuit, bilateral or even unilateral lesions in this region usually cause devastating effects on memory and learning.

The current case exhibited some singularities. Although the stroke was unilateral (left-sided lesion), both verbal and visual deficits of EM were observed. This is unusual since memory function specializes with hemispheric function: the left hemisphere is more related to verbal components of memory, while the right hemisphere mediates visuospatial features of memory. Also, the functional impairment in JE's everyday life after the stroke was highly severe. This is an uncommon finding because severe functional impairment in MTL lesions is more probable as a consequence of bilateral lesions as opposed to unilateral ones. Moreover, although isolated and stable memory impairment that presents acutely may be permanent, some patients experience changes over time. As expected for typical patients with MTL amnesia, JE also presented preserved insight, mild or absent retrograde amnesia and lack of confabulation. Furthermore, no semantic memory impairment was observed. Although semantic memory is a component of declarative memory along with EM, there has been a debate regarding the extent to which the encoding of new semantic information is dependent solely on the MTL structures, suggesting that semantic memory acquisition may differ in some manner compared to EM.^7^ Indeed, some evidence based on patients with MTL lesions has shown acquisition of new semantic information, in spite of major difficulty.^15^

This case shows that strategic infarct in the MTL structures results in features of EM impairment with preservation of other cognitive functions, followed by severe functional impairment. The case serves as an important example of brain structure-function correlation, a fundamental cornerstone of cognitive neurology and neuropsychology.
